# Blastocyst complementation-based rat-derived heart generation reveals cardiac anomaly barriers to interspecies chimera development

**DOI:** 10.1016/j.isci.2024.111414

**Published:** 2024-11-18

**Authors:** Shunsuke Yuri, Norie Arisawa, Kohei Kitamuro, Ayako Isotani

**Affiliations:** 1 Division of Biological Science, Graduate School of Science and Technology, Nara Institute of Science and Technology, 8916-5 Takayama-cho, Ikoma, Nara 630-0192, Japan; 2 Laboratory of Experimental Animals, Research Institution, National Center for Geriatrics and Gerontology, 7-430 Morioka-cho, Obu, Aichi 474-8511, Japan; 3 Life Science Collaboration Center (LiSCo), Nara Institute of Science and Technology, 8916-5 Takayama-cho, Ikoma, Nara 630-0192, Japan

**Keywords:** Health sciences, cardiovascular medicine, biological sciences

## Abstract

The use of pluripotent stem cells (PSCs) to generate functional organs via blastocyst complementation is a cutting-edge strategy in regenerative medicine. However, existing models that use this method for heart generation do not meet expectations owing to the complexity of heart development. Here, we investigated a Mesp1/2 deficient mouse model, which is characterized by abnormalities in the cardiac mesodermal cells. The injection of either mouse or rat PSCs into Mesp1/2 deficient mouse blastocysts led to successful heart generation. In chimeras, the resulting hearts were predominantly composed of rat cells; however, their functionality was limited to the embryonic developmental stage on day 12.5. These results present the functional limitation of the xenogeneic heart, which poses a significant challenge to the development in mouse–rat chimeras.

## Highlights

• Mesp1/2-deficient (DKO) mice provide a niche for heart ventricular formation
• Mouse ESCs rescue the lethal phenotype of cardiac deficiency in Mesp1/2-DKO mice
• The generated rat heart predominantly originates from the injected rat ESCs
• Rat ESCs rescue the lethal phenotype of Mesp1/2-DKO until E12.5 but fail by E14.5

## Graphical abstract

**Figure:**
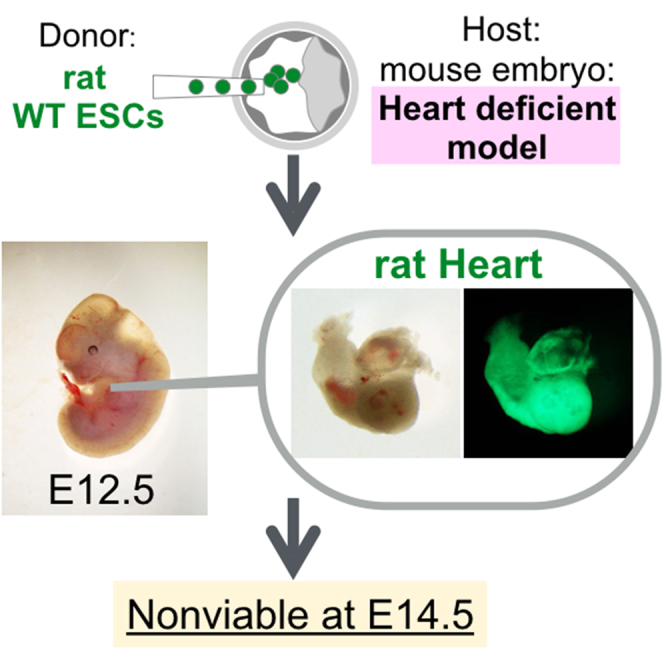

## Introduction

The mammalian heart is the first organ to form during embryonic development. It comprises four chambers, namely, the left atrium, right atrium, left ventricle, and right ventricle, which function as pumps to circulate blood and oxygen throughout the body. Each chamber comprises three tissue layers: the epicardium, myocardium, and endocardium. The myocardium is comprised of various cell types, including cardiomyocytes, fibroblast-like cells, vascular endothelial cells, smooth muscle cells, and pericytes.^1^^,^^2^^,^^3^ The epicardium is primarily comprised of epicardial cells,^4^ whereas the endocardium is comprised of endocardial endothelial cells.^5^ The adult mammalian heart is not regenerative owing to the limited proliferative capacity of the adult cardiomyocytes; therefore, ischemic heart disease is the leading cause of death worldwide.^6^ The ultimate treatment for end-stage heart failure is heart transplantation, and 4,000–5,000 cases of heart transplantation are performed annually; however, the demand is far higher, and donor organs are limited.^7^ Although the xenotransplantation of genetically modified porcine hearts into humans may offer a breakthrough solution to the global organ shortage problem, the associated immunologic issues are yet to be resolved.^8^

The development of human induced pluripotent stem cells (iPSCs) has led to global advancements in the effort to create functional organs from iPSCs. Consequently, the blastocyst complementation (BC) method has been used to produce primarily PSC-derived organs in animals. The BC method involves the injection of PSCs into fertilized eggs of organ-deficient animals at the blastocyst stage. The PSCs populate the defective developmental organ niche and produce PSC-derived organs. Following its initial application to generate lymphocytes,^9^ the intraspecies BC method has since been successfully used to produce various organs such as kidney, liver, lung, hematoendothelial tissues, thyroid, and forebrain.^10^^,^^11^^,^^12^^,^^13^^,^^14^^,^^15^^,^^16^^,^^17^^,^^18^ In contrast, the use of the interspecies BC method, which combines mouse and rat models, has not been widely reported; nevertheless, organs such as the pancreas, thymus, kidneys, germ cells, and lungs have been successfully generated using this method.^19^^,^^20^^,^^21^^,^^22^^,^^23^^,^^24^^,^^25^ Mouse PSCs that were used to generate the heart using the BC method rescued heart defects in both Id1/Id3 double knockout (DKO) mouse embryos and Nkx2.5-Cre/Rosa26-loxP-stop-loxP-DTA mouse embryos; however, the generated heart was composed of a mixture of PSC-derived and mutant cells.^26^^,^^27^ Similarly, rat PSCs restored the development of heart-deficient mice in Nkx2.5-KO embryos; however, these hearts consisted of cardiomyocytes derived from both mouse and rat cells.^28^ Therefore, heart-deficient models using the BC method have been deemed inadequate for generating hearts primarily from PSCs.

During the development of the mouse heart, the cardiac mesoderm forms along the primitive streak in the posterior part of the embryo by embryonic day 6.5 (E6.5), marking the initial sign of heart development. By E7.0, the mesodermal cells migrate to the anterior part of the embryo and form the first heart field (FHF). The FHF cells generate a cardiac crescent that eventually differentiates into the left ventricle and parts of the atria. Subsequently, the second heart field (SHF) is formed by the migrating mesodermal cells along the dorsal and medial sides of the cardiac crescent. The SHF cells contribute to the formation of the right ventricle, parts of the atria, and outflow tract.^29^ The juxta-cardiac field (JCF) cells are located rostral to the cardiac crescent and contribute to part of the myocardium of the left ventricle and epicardium.^30^ Additionally, cardiac neural crest cells migrate from the dorsal neural tube through the pharyngeal arches to the heart, contributing to the nervous system and smooth muscle cells within the aorta and pulmonary artery.^31^ Heartbeat is detected approximately at E8.0, and contraction of the cardiac muscle cells enables blood and nutrient circulation for the developing embryo. At approximately E11.5, vascular remodeling occurs, leading to the construction of the coronary arteries; additionally, the atria, ventricular septum, and valves begin to form. By E14.5, these structures mature into a four-chambered heart.^29^

Mesp1 is a transcription factor with a bHLH motif. It is highly expressed in the mouse embryo in the early mesodermal cells between E6.5 and E6.75 stages; however, after E7.0, its expression rapidly decreases. Mesp1-KO mice exhibit several morphogenetic abnormalities in the heart, leading to mortality before E10.5.^32^^,^^33^ During somitogenesis, Mesp1 rescues the function of Mesp2, which is encoded by a gene from the same family, indicating that Mesp1 and Mesp2 play a similar function during embryonic mesodermal development.^34^ Mesp2-KO mice exhibit inhibited the segmentation of somites, resulting in fused vertebrae and death within 20 min of birth.^35^^,^^36^ In Mesp1/2-DKO mice, the heart, somites, and gut fail to develop, resulting in death at approximately E9.5. Aggregation chimera analysis of Mesp1/2-DKO embryos with wild-type (WT) embryos showed that at E8.5 or E9.5, Mesp1/2-DKO cells were nearly absent in the heart and were compensated for by WT cells; however, analysis was not performed at a later stage.^37^

In this study, we aimed to generate rat PSC-derived hearts using the interspecies BC method. To establish an organ-deficient model in which the BC method-based PSC-derived organs can develop, we investigated both Mesp1-KO or Mesp1/2-DKO models and ascertained which of the two was more suited for this study. We clarified the conditions for successful heart generation using the reverse-BC (rBC) method, which yields chimeras derived from mutant and WT cells with high efficiency.^25^ Then, we generated hearts through rat cell complementation in the Mesp1/2-DKO mouse model using the interspecies BC method.

## Results

### Mesp1-KO model is not a suitable heart-deficient model

**Figure 1:**
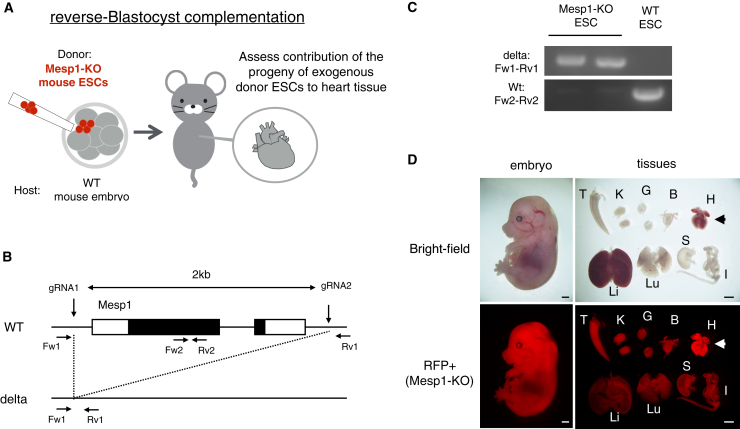
Analysis of the Mesp1-KO model in the heart with reverse blastocyst complementation (rBC) method (A) Schematic of reverse-blastocyst complementation (rBC) method. Mesp1-knockout (KO) embryonic stem cells (ESCs) expressing red fluorescent protein (RFP) were injected into the wild type (WT) embryo. Mesp1-KO ESCs+ WT chimeras were dissected to assess the contribution of the progeny of exogenous donor ESCs to heart tissue. (B) Strategy for generation of Mesp1-KO model. Both gRNA1 and gRNA2 were used to remove exon1 and exon 2 of the *Mesp1* gene. Fw1-Rv1 primer set was used to detect the deletion of the *Mesp1* gene. Fw2-Rv2 primer set was used to detect the WT allele of the *Mesp1* gene. (C) Genotype of Mesp1-KO, Mesp1 heterozygous (Het), and Mesp1 WT ESCs. (D) Representative embryo and organs derived from RFP-expressing Mesp1-KO ESCs at E14.5. (T; Tail, K: Kidney, G: Gonad, B: Bladder, H: Heart, Li: Liver, Lu: Lung, S: Stomach, I: Intestine) Scale bars, 1 mm.

Mesp1-KO mice exhibit cardiac dysplasia, which is characterized by atypical cardiac morphogenesis that results from delayed mesodermal cell migration.^33^ Based on the assumption that WT cells would have a competitive advantage over Mesp1-KO cells, we hypothesized that creating chimeric mice with both Mesp1-KO and WT cells would generate hearts that are fully comprised of WT cells, offering an ideal outcome for the BC method. To test this hypothesis, we used the rBC method and analyzed the resulting chimeras (donor: Mesp1-KO ESCs; host: WT embryos) (Figure 1A). Guide RNAs (gRNAs) were designed to flank exons 1 and 2 of the *Mesp1* gene to generate Mesp1-KO embryonic stem cell (ESC) lines expressing red fluorescent protein (RFP) (Figures 1B and 1C). Two ESC lines with unique mutations were used to generate the chimeras (Figure S1A). These chimeric embryos were generated by injecting Mesp1-KO ESCs into WT embryos at E2.5. They were analyzed at E14.5 (Table 1). Contrary to the assumption, we identified RFP-expressing Mesp1-KO cells in cardiac tissue and several other organs (Figure 1D). Further evaluation of the presence of the Mesp1-KO-derived RFP+ cells in the chimeric tissues using flow cytometry showed that their contribution to heart tissue was the same as that in most other tissues (Figure S1B). Consequently, the Mesp1-KO model was deemed not suitable as a heart-deficient BC method model.

**Table 1:** Result of ESC injection with the rBC method (E8.5 and E14.5 analysis)

ESC line	Analysis Stage	Transplantation	Implantation	Live embryos	RFP+ chimera
Mesp1-KO (1H)	E14.5	41	24	17	11
Mesp1-KO (2G)	E14.5	42	34	20	13
WT ESC	E14.5	74	42	23	13
WT ESC	E8.5	37	25	14	11
Mesp1/2-DKO (2G)	E8.5	61	36	19	11
Mesp1/2-DKO (3F)	E8.5	42	25	12	5
Mesp1/2-DKO (2G)	E14.5	128	64	28	21
Mesp1/2-DKO (3F)	E14.5	107	40	23	19

### Wild-type cells dominate heart tissue in the Mesp1/2-DKO model at E8.5

**Figure 2:**
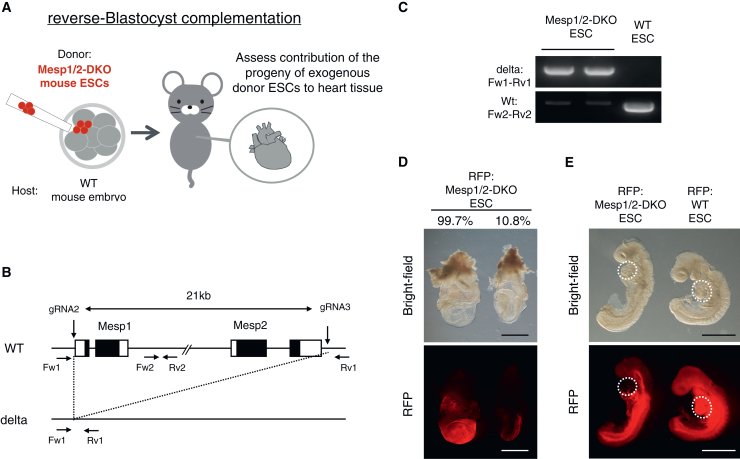
Analysis of Mesp1/2-DKO model using rBC method at E8.5 (A) Schematic of reverse-blastocyst complementation (rBC) method. Mesp1/2-double knockout (DKO) embryonic stem cells (ESCs) expressing red fluorescent protein (RFP) were injected into the wild type (WT) embryo. Mesp1/2-DKO ESCs+WT chimeras were dissected to assess the contribution of the progeny of exogenous donor ESCs to heart tissue. (B) Strategy for the generation of the Mesp1/2-DKO model. Both gRNA2 and gRNA3 were used to remove *Mesp1* and *Mesp2* genes. Fw1-Rv1 primer set was used to detect deletion of both *Mesp1* and *Mesp2* genes. Fw2-Rv2 primer set was used to detect no deletion of *Mesp1* and *Mesp2* genes. (C) Genotype of Mesp1/2-DKO and WT ESCs. (D) Representative images of embryos derived from RFP-expressing Mesp1/2-DKO ESCs at E8.5. Mesp1/2-DKO cells with higher chimerism showed abnormal shape. Scale bars, 1 mm. (E) Representative image of embryos derived from RFP-expressing Mesp1/2-DKO and WT ESCs at E8.5. RFP+ cells are not detected in the Mesp1/2-DKO chimera heart. Scale bars, 1 mm.

To further explore the heart-deficient model for the BC method, we analyzed the Mesp1/2-DKO model using the rBC method based on previous studies^37^ (donor: Mesp1/2-DKO ESCs; host: WT embryos) (Figure 2A). *Mesp1* and *Mesp2* genes are located on the same chromosome and separated by approximately 21 kb. We designed two gRNAs to flank these genes to delete the entire region encompassing both genes (Figure 2B) and established two Mesp1/2-DKO ESC lines, each with a different mutation (Figures 2C and S2A). To investigate the contribution of Mesp1/2-DKO cells to heart development, we analyzed chimeric embryos containing Mesp1/2-DKO and WT cells on E8.5 (Table 1). In the chimeras with high Mesp1/2-DKO cell contribution rates (99.7%), the embryonic structure was unclear, and extensive accumulation of cell layers was observed in the extraembryonic region. This phenotype is similar to that of the Mesp1/2-DKO embryos at E8.5–E9.0 (Figure 2D).^37^ In contrast, in chimeras with low Mesp1/2-DKO cell contribution rates (10.8%), the heart structure was confirmed but the Mesp1/2-DKO cells contributed minimally to the heart (Figure 2E). This indicates that the heart tissue in the Mesp1/2-DKO model was predominantly composed of WT cells.

### Heart function was preserved until the postnatal stage in Mesp1/2-DKO chimeras

**Figure 3:**
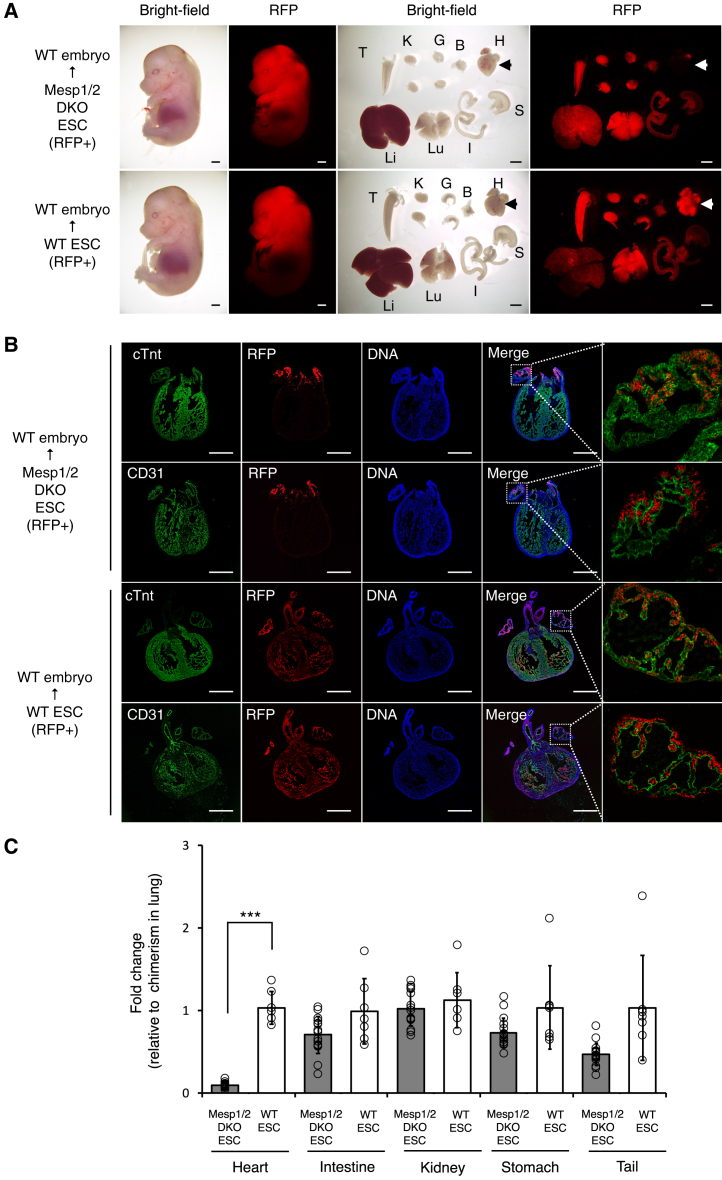
Analysis of Mesp1/2-DKO model using rBC method at E14.5 (A) Representative image of embryos and organs derived from red fluorescent protein (RFP)-expressing Mesp1/2-double knock out (DKO) ESCs at E14.5. (T; Tail, K: Kidney, G: Gonad, B: Bladder, H: Heart, Li: Liver, Lu: Lung, S: Stomach, I: Intestine) Scale bars, 1 mm. (B) Representative immunostaining image of cTnt and CD31 in the heart of Mesp1/2-DKO ESCs+WT or WT ESCs+WT chimera. Scale bars, 500 μm. (C) Flow cytometry analysis of tissues from the Mesp1/2-DKO ESCs+WT or WT ESCs+WT chimeras. The fold change shows that the chimerism of each tissue (heart, intestine, kidney, stomach, and tail) was divided into the chimerism of the lung in the chimeras. All values are expressed as mean ± standard deviation from at least triplicate experiments (*n* = 16 in Mesp1/2-DKO chimera; *n* = 9 in WT chimera, ∗∗∗: *p* < 0.01).

Next, we characterized the distribution of Mesp1/2-DKO cells in later-stage embryos to elucidate their contribution to the heart (Table 1). Consistent with the findings at E8.5, Mesp1/2-DKO cells were minimally incorporated into the cardiac tissue on E14.5 but were present in various other organs (Figure 3A). A detailed examination of the Mesp1/2-DKO (RFP+) cells within the cardiac component cells showed partial contribution to the cTnt+ cardiomyocytes and CD31^+^ cardiac endothelium, particularly in the atrial region (Figure 3B). Using flow cytometry, we compared the contribution rate of Mesp1/2-DKO cells across various organs. Their incorporation into cardiac tissue was significantly lower than that observed in other organs as the majority of cardiac cells were of WT origin (Figure 3C). In contrast to that observed at E8.5, embryos with WT cell contribution rates <20% were not observed at E14.5 (Figure S2B). Collectively, these findings indicate that the Mesp1/2-DKO model developed in this study necessitates the contribution of >20% of WT cells to rectify cardiac anomalies; hence, it predominantly functions as a model of cardiac tissue deficiency.

**Figure 4:**
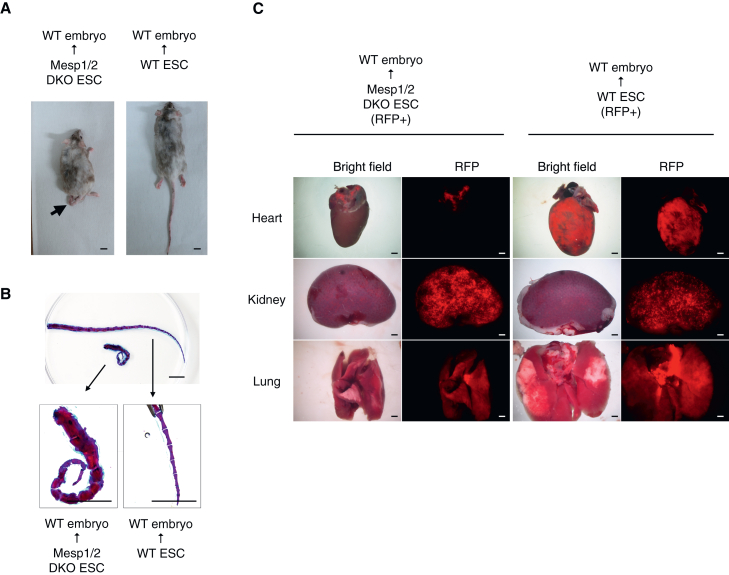
Analysis of the Mesp1/2-DKO model using the rBC method at 8 weeks (A) Representative image of chimera mice derived from Mesp1/2-double knock out (DKO) ESCs and WT cells or WT ESCs and WT cells at 8 weeks old. Mesp1/2-DKO ESCs+WT chimeras show clear shortened tail morphology (arrow). Scale bars, 1 cm. (B) Representative image of Alcian blue and Alizarin red staining of the tail from Mesp1/2-DKO ESCs+WT or WT ESC+WT chimeras. Scale bars, 1 cm. (C) Macroscopic images of the heart, kidney, and lung of chimera mice from red fluorescent protein (RFP) expressing Mesp1/2-DKO ESCs+WT or RFP expressing WT ESCs+WT. (*n* = 4 chimeras from Mesp1/2-DKO ESC or WT ESC injection) Scale bars, 1 cm.

Furthermore, analysis was performed at the postnatal stage to determine whether the Mesp1/2-DKO ESCs+WT chimeras exhibited cardiac phenotypic abnormalities after birth (Table 2). As a result, Mesp1/2-DKO ESCs+WT chimeras were viable and displayed normal motility for at least 8 weeks (Video S1). Notably, a consistent phenotype of curled and shortened tails was observed in the Mesp1/2-DKO ESCs+WT chimeras (Figure 4A). Skeletal staining with Alcian blue and Alizarin red showed that the abnormality in tail morphology of Mesp1/2-DKO ESCs+WT chimeras was due to a reduced number of segments and disrupted segment boundaries compared with that of the skeletons derived from WT ESCs+WT chimeras (Figure 4B). Fluorescence imaging of the heart revealed that Mesp1/2-DKO (RFP+) cells remained localized in the atrial region but were absent from the ventricular region in the Mesp1/2-DKO ESCs+WT chimeras. This mirrors the findings at E14.5. In contrast, the ESC-derived cells (RFP+) were uniformly distributed in all cardiac tissues in WT ESCs+WT chimeras (Figure 4C). Additionally, RFP+ cells were detected in other organs, including kidneys and lungs, in both Mesp1/2-DKO ESCs+WT chimeras and WT ESCs+WT chimeras (Figures 4C and S2C). Taken together, these results suggest that Mesp1/2-DKO cells could not contribute to ventricular tissue following chimera formation with WT cells and that cardiac function is maintained until the postnatal stage even when the Mesp1/2-DKO cells contribute to atrial cells.

**Table 2:** Result of ESC injection with the rBC method (8 weeks old analysis)

ESC line	Analysis Stage	Transplantation	Live pups	RFP+ chimera
WT ESC	8 weeks old	10	5	4
Mesp1/2-DKO (2G)	8 weeks old	40	6	4

Video S1. The movement of the chimeras from Mesp1/2-DKO ESCs at P56

### Intraspecies blastocyst complementation method generated embryonic stem cell-derived heart in the Mesp1/2-DKO model

**Figure 5:**
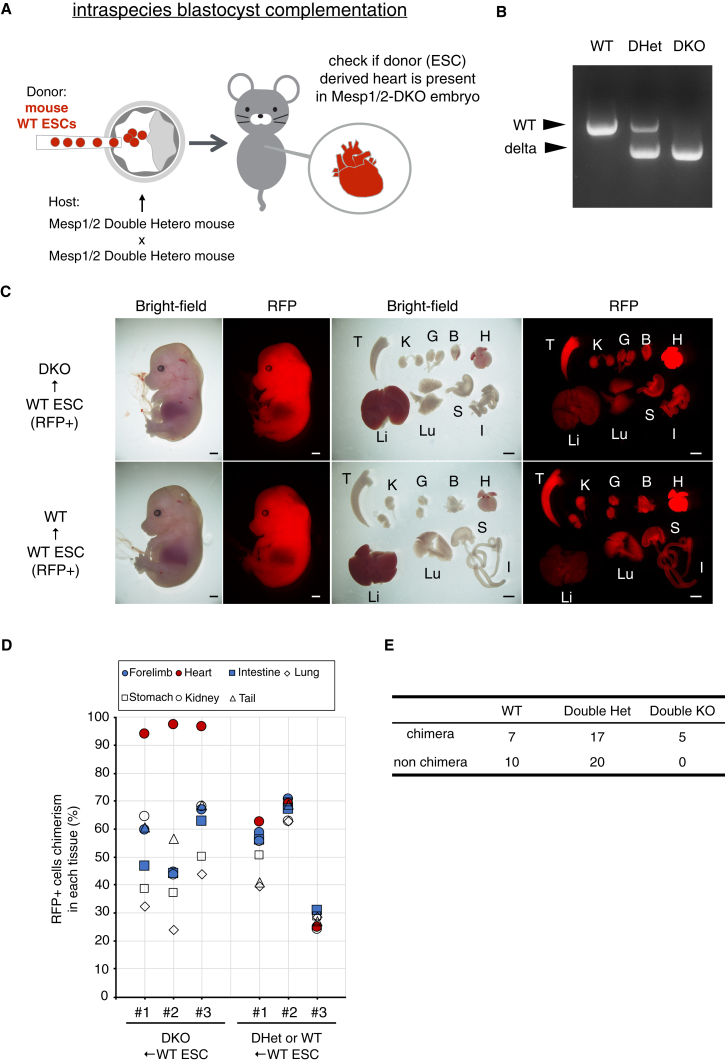
Intraspecies BC method-generated Mesp1/2-DKO mouse model (A) Schematic of intraspecies blastocyst complementation (BC) method. Red fluorescent protein (RFP) expressing mouse WT ESCs were injected into the embryos obtained by crossing Mesp1/2 double heterozygous (DHet) male and female mice. WT ESCs+Mesp1/2-DKO or WT ESCs+non-Mesp1/2-DKO chimeras were dissected at E14.5. (B) Genotype of Mesp1/2-DKO, Mesp1/2-DHet, and WT embryos. RFP negative population was sorted to remove WT cells derived from injected ESCs (RFP+). See genotyping strategy in Fig.S3E. (C) Representative image of embryos and organs derived from WT ESCs (RFP+) +Mesp1/2-DKO or WT ESCs (RFP+) +non-Mesp1/2-DKO chimeras. Chimeras were viable in the Mesp1/2-DKO genotype, and the RFP-expressing heart was observed. (T; Tail, K: Kidney, G: Gonad, B: Bladder, H: Heart, Li: Liver, Lu: Lung, S: Stomach, I: Intestine) Scale bars, 1 mm. (D) Flow cytometry analysis of organs (forelimb, heart, intestine, lung, stomach, kidney, and tail) in chimeras from WT ESCs (RFP+) +Mesp1/2-DKO or WT ESCs (RFP+) +non-Mesp1/2-DKO. The heart is almost composed of RFP-expressing WT cells in all embryos of the Mesp1/2-DKO genotype (*n* = 3). (E) Results of intraspecies blastocyst complementation by injecting mouse ESCs into blastocysts obtained by crossing Mesp1/2-DHet with Mesp1/2-DHet. Chimeras with almost 100% ESC contribution were removed from the genotype analysis owing to the difficulty of sorting RFP negative population.

To evaluate the Mesp1/2-DKO model using the BC method, Mesp1/2 double heterozygous (DHet) mice were generated from zygotes using the same gRNAs as those utilized in the rBC method, through the electroporation method (Figure S3A). Eight pups were obtained from 88 electroporated embryos. Of these, seven exhibited mutations in the gRNA2 region and three in the gRNA3 region; however, one showed a successful 21-kb deletion (Figures S3B and S3C), which was confirmed by sequencing (Figure S3D). By interbreeding the Mesp1/2-DHet males and females, we obtained blastocysts and injected RFP-expressing mouse WT ESCs (donor: mouse WT ESCs; host: Mesp1/2-DKO or non-Mesp1/2-DKO embryos) (Figure 5A) (Table 3). As the ESC-derived mouse WT cells were present in the chimera, the tail or forelimb was dissociated and sorted to remove the RFP positive WT cells originating from WT ESCs before genotyping (Figure S3E). As a result, genotype analysis identified DKO genotype embryos in the WT cell-reconstituted chimeras (Figure 5B). Analysis of organs from WT ESCs+Mesp1/2-DKO chimeras showed normal cardiac morphology similar to that of WT ESCs+non-Mesp1/2-DKO chimeras (Figure 5C). Flow cytometry analysis indicated that WT (RFP+) cells were predominantly present in the cardiac tissues in WT ESCs+Mesp1/2-DKO chimeras, similar to the results observed in the rBC experiment (Figure 5D). Additionally, the DKO genotype was not observed in non-chimeric embryos in which WT ESCs did not contribute, indicating that WT ESCs rescued the lethal phenotype of cardiac deficiency in Mesp1/2-DKO mice (Figure 5E). These findings imply that the Mesp1/2-DKO mouse model established here exhibited cardiac deficiency comparable to that of the rBC method-generated model.

**Table 3:** Result of WT ESC injection with the intraspecies BC method

ESC line	Transplantation	implantation	live embryos	RFP+ chimera
WT ESCs	243	137	72	42

### Generated rat embryonic stem cell-derived heart in Mesp1/2-DKO mouse model functioned until E12.5

**Figure 6:**
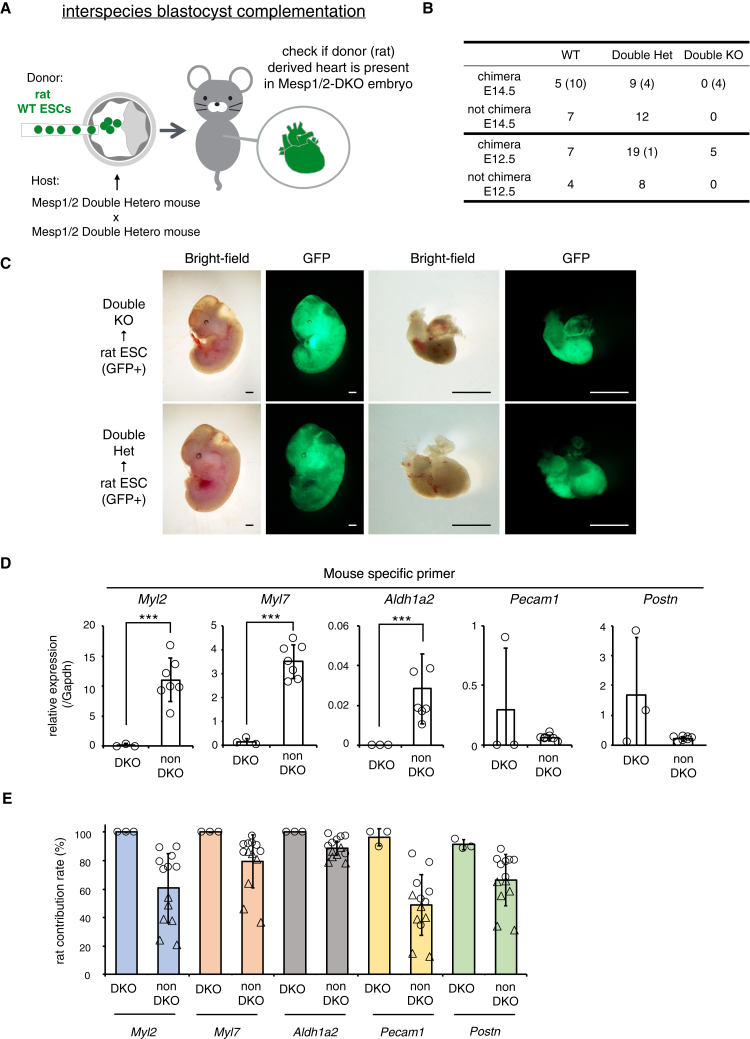
Interspecies BC method for generating Mesp1/2-DKO mouse model (A) Schematic of interspecies blastocyst complementation (BC) method. Green fluorescent protein (GFP) expressing rat ESCs were injected into the embryos obtained by crossing Mesp1/2 double heterozygous (DHet) male and female mice. Rat ESCs+Mesp1/2-DKO or rat ESCs+non-Mesp1/2-DKO chimeras were dissected at E14.5 and E12.5. (B) Results of interspecies blastocyst complementation by injecting rat ESCs into blastocysts obtained by crossing Mesp1/2-DHet with Mesp1/2-DHet. The numbers in parentheses indicate resorbed embryos. (C) Representative images of embryos and hearts derived from chimeras from rat ESCs+Mesp1/2-DKO or rat ESCs+non-Mesp1/2-DKO. The chimeras were viable in the Mesp1/2-DKO genotype, and GFP expressing heart was observed. Scale bars, 1 mm. (D) Quantitative real-time PCR results for mouse *Myl2*, *Myl7*, *Aldh1a2*, *Pecam1,* and *Postn*. Data were normalized to mouse *Gapdh* expression levels. Samples were extracted from the hearts of chimeras. All values are expressed as mean ± standard deviation from at least triplicate experiments (DKO: *n* = 3 rat chimeras, non-DKO: *n* = 7 rat chimeras). ∗∗∗: *p* < 0.01; unpaired two-tailed Student’s t test. (E) Variation in the presence of rat cells in each cardiac tissue component. Mostly, rat *Myl2*, *Myl7*, *Aldh1a2*, *Pecam1*, and *Postn* were detected compared with mouse genes in the rat Mesp1/2-DKO chimera genotype. Open circles in the non-Mesp1/2-DKO genotype indicate the results obtained from higher rat chimerism in the tail (28–67%). Open triangles in the non-Mesp1/2-DKO genotype showed the results obtained from lower rat chimerism in the tail (6–12%). All values are expressed as mean ± standard deviation from at least triplicate experiments (*n* = 3 in DKO; *n* = 7 in non-DKO with higher rat chimerism; *n* = 6 in non-DKO with lower rat chimerism).

In rat ESCs+mouse WT chimeras, rat cells readily contributed to more than 20% of the cardiac tissue at E14.5 (Figure S4A). Therefore, we hypothesized that generating interspecies chimeras using rat ESCs in the Mesp1/2-DKO mouse model could potentially remedy cardiac deficiency, at least until E14.5. To determine whether rat cells were capable of heat generation in the interspecies BC method using the Mesp1/2-DKO mouse model, we injected rat ESCs (green fluorescent protein (GFP)+) into the blastocysts obtained by interbreeding the Mesp1/2-DHet males and females (donor: rat ESCs; host: Mesp1/2-DKO or non-Mesp1/2-DKO embryos) (Figure 6A) (Table 4). The results showed that no live rat chimeras with the Mesp1/2-DKO genotype were recovered on E14.5 (Figure 6B). On the other hand, rat ESCs+rat WT chimeras generated by injecting rat ESCs into rat WT embryos, which showed higher chimerism of the rat ESC-derived cells, were obtained even at E16.5 (Figure S4B). This result indicated that the injected rat ESCs did not adversely affect heart development in rats. Typically, Mesp1/2-DKO mice die approximately at E9.5; however, the resorbed rat ESCs+Mesp1/2-DKO chimeras clearly progressed beyond this stage (Figure S4C), suggesting that the rat cells might have partially compensated the cardiac defect due to Mesp1/2 gene deficiency. Based on these findings, we examined rat ESCs+Mesp1/2-DKO chimeras on E12.5. At E12.5, we identified live rat chimeras with the Mesp1/2-DKO genotype exhibiting GFP-positive hearts and observable heartbeat (*n* = 4) (Figures 6B and 6C; Video S2). This suggests that the cardiac defects in Mesp1/2-DKO mice could be ameliorated by using rat cells and that the rat-derived hearts were functional up to E12.5. To examine whether rat cells could complement the heart tissues in the Mesp1/2-DKO mouse, we immunostained with cardiomyocyte (cTnt) and cardiac endothelium (CD31). The results showed that cTnt+ and CD31^+^ cells were mostly composed of GFP+ rat cells (Figure S4D). Further quantitative analysis of the rat-derived hearts from the Mesp1/2-DKO model was performed using RT-PCR with mouse specific primers. The RT-PCR result of atrial cardiomyocyte marker *Myl2*, ventricular cardiomyocyte marker *Myl7*, epicardial marker *Aldh1a2*, pan-endothelial marker *Pecam1*, and fibroblast-like cell marker *Postn*^38^^,^^39^^,^^40^ showed that *Myl2*, *Myl7*, and *Aldh1a2* were minimally detected in the mouse cell population within the cardiac tissues of rat ESCs + Mesp1/2-DKO chimeras (Figure 6D). Conversely, *Pecam* and *Postn* expression was higher in the mouse cell population of the cardiac tissues in the rat ESCs+Mesp1/2-DKO chimeras than in those of the rat ESCs+non-Mesp1/2-DKO chimeras (Figure 6D). Furthermore, when assessing the proportional contribution of mouse and rat cells to each cardiac tissue component, we observed that ventricular and atrial cardiomyocytes and epicardial, endothelial, and fibroblast-like cells were predominantly composed of rat cells in the rat ESCs+Mesp1/2-DKO chimeras (Figure 6E). Notably, the rat ESCs+non-Mesp1/2-DKO chimeras, which showed higher levels of rat chimerism in the tail, exhibited a more prominent expression of rat genes, such as *Myl2*, *Myl7*, *Aldh1a2*, and *Postn*, compared to mouse genes. However, the percentage of rat cells in these cell types was lower than that in the hearts of rat ESCs+Mesp1/2-DKO chimeras (Figure 6E). These results indicate that rat cells generated mostly rat-derived hearts in the Mesp1/2-DKO model, but the rat-derived hearts lost their function by E14.5.

**Table 4:** Result of rat ESC injection with the interspecies BC method

Analysis stage	Transplantation	Implantation	embryos	GFP+ chimera
E12.5	261	141	46	32
E14.5	322	175	59	32

Video S2. The heartbeat of the rat chimera in the Mesp1/2-DKO model

### Heart with high rat contribution in mice was not functional after E12.5

**Figure 7:**
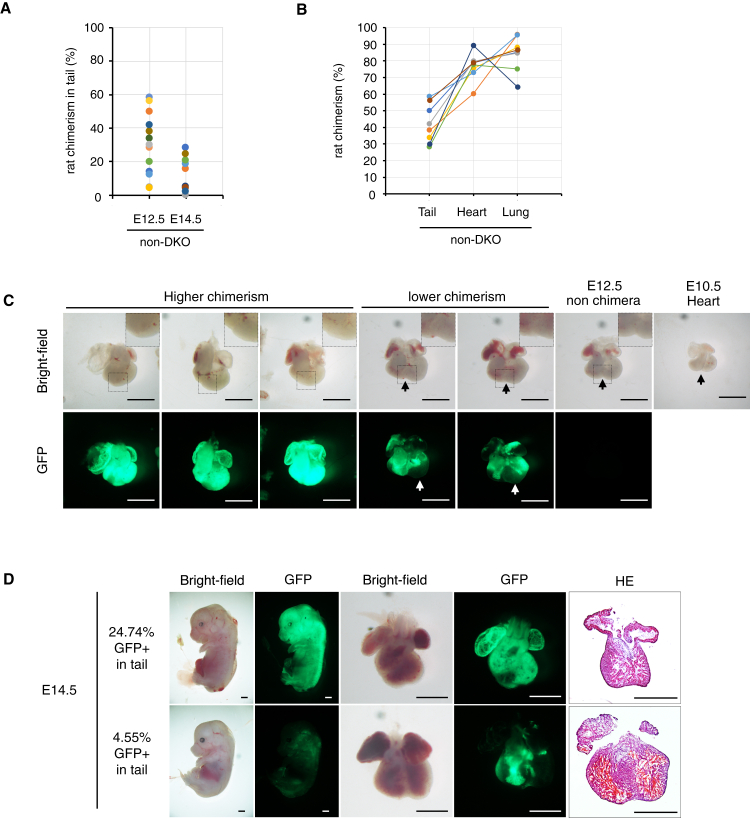
Analysis of mouse-rat chimera in non-Mesp1/2-DKO mouse (A) Flow cytometry analysis of tail of rat ESCs+non-Mesp1/2-DKO chimeras at E12.5 and E14.5. (*n* = 17 at E12.5, *n* = 13 at E14.5). (B) Flow cytometry analysis of tail, heart, and lung of rat ESCs+non-Mesp1/2-DKO chimeras at E12.5 (*n* = 8). (C) Representative heart images of higher rat chimerism (44.2%, 44.2%, and 54.7% in tail) and lower rat chimerism (11.7%, 8.3% in tail) at the E12.5 stage. No rat contribution was observed at E12.5 and E10.5 stages in the non-Mesp1/2-DKO genotype. The arrow indicates the interventricular sulcus. The white arrow indicates the loss of rat cells' contribution to the left ventricle. (Scale bar: 1 mm). (D) Representative embryo, heart, and HE staining images of rat chimera in the non-Mesp1,2-DKO genotype (Scale bar: 1 mm).

In the rat, ESCs+non-Mesp1/2-DKO chimeras, numerous resorbed embryos were observed at E14.5 (Figure 6B). When comparing rat chimerism at E12.5 and E14.5, none of the chimeras had more than approximately 30% rat cells in the tail at E14.5 (Figure 7A). When rat chimerism in the tail was between 30 and 60% at E12.5, rat chimerism in the heart was approximately 60–90% (Figure 7B). This suggests that the hearts of chimeras with over 30% rat chimerism in the tail were predominantly comprised of rat cells, which aligns with the results of the RT-PCR analysis (Figure 6E). Therefore, we hypothesized that the resorbed rat chimeras also exhibited heart abnormalities because of the high contribution from rat cells in the heart. Recently, we reported that rat-derived lungs in mice may retain rat-specific developmental timing, which is related to lung functionality at P0^25^. To determine whether hearts with higher rat chimerism also exhibited developmental delays, we compared heart morphology at E12.5 with that at E10.5. As a result, the hearts with higher rat contributions were not identical to the heart at E10.5 but distinctly exhibited abnormal shapes with the typical disruption of the interventricular sulcus at E12.5 (Figure 7C). On the other hand, lung epithelial cells in chimeras with higher rat chimerism of 33.2–68.9% in the tail exhibited growth retardation at E12.5 (Figure S5). The rat cells were often absent in the left ventricle in the rat chimeras with lower levels of rat chimerism (Figure 7C). Furthermore, embryos with tail chimerism of approximately 25% at E14.5 showed signs of resorption with abnormal heart morphology (Figure 7D). These results indicate that even in the non-heart deficient model, the heart tissue of mouse–rat chimeras may be predominantly composed of rat cells, and mouse-rat chimeras with high rat contribution may be lethal owing to cardiac abnormalities that manifest between E12.5 and E14.5 rather than developmental delay.

## Discussion

In the present study, we used the rBC method to assess the efficacy of the Mesp1/2-DKO model in generating PSC-derived hearts. The findings elucidated the conditions required for heart generation. Subsequently, successful heart generation was achieved using rat cells in a mouse model that lacked a cardiac niche via the BC method.

Despite previous findings on cardiac morphological abnormalities in Mesp1-KO mice,^33^ analysis of Mesp1-KO ESCs+WT chimeras showed that Mesp1-KO cells contribute to the heart in tandem with WT cells. This suggests that the Mesp1-KO mouse model is not an ideal heart-deficiency model for the BC method. This conclusion is supported by recent studies that indicate that Mesp2 compensates for the absence of Mesp1, and dose dependency of Mesp1 and Mesp2 is critical during early mesoderm formation.^41^ Thus, a heart-deficiency model requires the complete functional loss of both *Mesp1* and *Mesp2* genes. Moreover, analysis of the Mesp1/2-DKO model using the rBC method indicates that the Mesp1/2-DKO primarily models cardiac ventricular defects. This finding is consistent with the results of previous studies that used aggregation methods.^37^ Analysis of Mesp1/2-DKO ESCs+WT chimeras at 8 weeks post-birth revealed a distinct tail abnormality, similar to a previous report.^42^ This unique phenotype indicated that although the Mesp1/2-DKO cells were outcompeted and eliminated by the WT cells during cardiac development, the Mesp1/2-DKO cells could coexist with and perturb the WT cells during somitogenesis. This suggests that Mesp1 and Mesp2 differ in function during cardiac development and somitogenesis. The observation that WT and mutant cells did not always engage in cell competition underscores the need for careful consideration when applying the KO model to the BC method.

Recent advancements have introduced a refined BC method for generating cardiac tissues using Nkx2.5-Cre and Tie2-Cre with Rosa26-LoxP-STOP-LoxP-DTA (R26-DTA) systems,^43^ where Nkx2.5-Cre induces cardiac deficiencies and Tie2-Cre induces vascular deficiencies. Both tissue types were successfully restored with PSCs using the intraspecies BC method. However, in the interspecies BC method using rat PSCs, only interspecies chimeras were restored in the Nkx2.5-Cre/R26-DTA model. This finding is consistent with the fact that the lethal phenotype of the Flk1-deficient model (which lacks vascular endothelial cells) was not rescued in the interspecies chimera after E10.5.^12^^,^^44^ This indicates the difficulties in achieving vascular complementation with rat cells in the mouse model. In the Nkx2.5-Cre/R26-DTA approach, interspecies chimeras were only present until E10.5. In contrast, interspecies chimeras were identified until E12.5 in the Mesp1/2-DKO model that was used in the current study; this is a discrepancy compared with prior observations. In the Mesp1/2-DKO model, rat cells complemented most of the cardiac tissues, including the cardiac vascular system. In contrast, in the Nkx2.5-Cre/R26-DTA model, only the cardiac tissues were complemented by rat cells; hence, they lack cardiac vascular evidence. This suggests potential incompatibilities in rat–mouse cell interactions during heart development, particularly between E10.5 and E12.5 stages. Another possibility is that the absence of rat cell complementation in non-cardiac tissues expressing Nkx2.5 in the Nkx2.5-Cre/R26-DTA system may lead to lethality after E10.5.

Consistent with earlier findings,^37^ our study demonstrated that the Mesp1/2-DKO model did not show atrial defects using the rBC method. However, our results revealed that using the interspecies BC method also led to the successful complementation of the atrial cardiomyocytes with rat cells in the Mesp1/2-DKO model. This difference between the intra- and inter-species BC methods indicates that rat cells may outperform mouse cells in the competitive environment of atrial cells. Notably, the rat cells even in the non-Mesp1/2-DKO genotype chimeras were more likely to contribute to the heart. This species-specific contribution pattern extended beyond the heart to other organs such as the mouse lungs and stomach, although rat cells contributed less to the mouse tail and kidneys.^25^^,^^45^ Additionally, in the rat cell-contributed heart, rat cells were often absent from the left ventricle, suggesting that rat cells may face challenges in competing with mouse cells for first heart field (FHF) cell lineages during the early mouse heart development. This suggests that rat cells have different competitive abilities depending on the tissue, potentially leading to unforeseen results in the interspecies BC method. Thus, gaining an understanding of species-specific cell competitive ability in different tissues is important and warrants future studies, given the critical role of normal cells in the niche of a deficient organ in the BC method. Furthermore, chimeras with higher rat contribution in the heart died at or before E14.5, suggesting that heart abnormalities are one of the factors contributing to the interspecies barrier in mouse-rat chimeras; therefore, methods need to be developed to overcome the rat contribution rate in the heart during this developmental period.

### Limitations of the study

In this study, the mechanisms underlying the preferential contribution of rat cells to mouse hearts, as well as the causes of the abnormalities observed in rat-derived hearts within the mouse environment, remain unclear. To elucidate these mechanisms, single-cell RNA sequencing (scRNA-seq) would be highly advantageous. Future studies employing scRNA-seq analysis in the heart and other tissues of mouse-rat chimeras or rat-complemented mouse chimeras could help identify the specific cell populations involved and clarify the molecular pathways contributing to the observed abnormalities. This would advance our understanding of interspecies chimerism and improve the success of functional organ generation through blastocyst complementation. Furthermore, understanding these mechanisms would offer valuable insights for addressing similar challenges in the application of interspecies blastocyst complementation in larger animals.

## Resource availability

### Lead contact

Further information and requests for resources and reagents should be directed to and will be fulfilled by the lead contact, Shunsuke Yuri (shunsukeyuri@ncgg.go.jp).

### Materials availability

Cell lines and mouse lines generated in this study are available with a material transfer agreement. Requests should be directed to the lead contact.

### Data and code availability

• Data: This study did not generate any unique dataset.
• Code: This study did not generate any original code.
• Additional information: All used software is listed in key resources table. Any additional information required to reanalyze the data reported in this article is available from the lead contact upon reasonable request.

## Acknowledgments

We thank the members of Isotani Laboratory for their helpful assistance and discussions. The LiSCO at the Nara Institute of Science and Technology (NAIST) and the core facility at the National Center for Geriatrics and Gerontology (NCGG) were instrumental in this study. We thank Dr. Ikawa (Osaka University) for kindly providing R01 ESCs. This study was supported by grants from the 10.13039/501100001691Japan Society for the Promotion of Science KAKENHI (Grant Numbers 23K18577 and 24K01948 to A.I. and 18K06031 and 22K06067 to S.Y.); KAC 40th Anniversary Research Grant to A.I.; the 10.13039/501100011746NOVARTIS Foundation (Japan) for the Promotion of Science to A.I.; and the 10.13039/100016985Foundation for Nara Institute of Science and Technology to S.Y. We also would like to thank Editage (www.editage.com) for English language editing.

## Author contributions

Conceptualization: S.Y. and A.I.; methodology: S.Y. and N.A.; validation: S.Y. and A.I.; formal analysis: S.Y. and N.A.; investigation: S.Y., N.A., and K.K.; writing–original draft: S.Y.; writing—review and editing: S.Y. and A.I.; visualization: S.Y. and N.A.; supervision: A.I.; funding acquisition: S.Y. and A.I.

## Declaration of interests

The authors declare no competing or financial interests.

## STAR★Methods

### Key resources table

**Table:** 

REAGENT or RESOURCE	SOURCE	IDENTIFIER
**Antibodies**		

Cardiac Troponin T Monoclonal Antibody (13-11) (dilution 1:100)	ThermoFisher Scientific	MA5-12960; RRID: AB_11000742
Human/Mouse/Rat CD31/PECAM-1 Antibody (dilution 1:100)	R&D systems	AF3628; RRID: AB_2161028
Goat Alexa Fluor 647 anti-mouse IgG (dilution 1:1000)	ThermoFisher Scientific	A21237; RRID: AB_1500743
Donkey Anti-Goat IgG H&L (Alexa Fluor® 647) preadsorbed (dilution 1:1000)	Abcam	ab150135; RRID: AB_2687955

**Chemicals, peptides, and recombinant proteins**		

CHIR99021	Axon	1386
CGP77675	Sigma	SML0314
PD0325901	Wako	162–25291
N2	ThermoFisher Scientific	17502048
B27	ThermoFisher Scientific	17504044
CARD HyperOva	Kyudo	1.0mL
Puromycin	Sigma	P9620
Hoechs33342	Dojindo	KV072
Guide-it™ Recombinant Cas9 (3 μg/μL)	TAKARA	632640
Opti-MEM	ThermoFisher Scientific	31985062
2.5% Trypsin	Nacalai Tesque	18172–94
Alcian blue Stain Solution (pH 2.5)	Nacalai Tesque	37154–15
Alizarin Red S	Nacalai Tesque	01303–52
NBT/BCIP Ready-to-Use	Nacalai Tesque	19880–84

**Critical commercial assays**		

Trizol reagent	ThermoFisher Scientific	15596026
SuperScript IV VILO master mix	ThermoFisher Scientific	11756050
GoTaq® Green Master Mix	Promega	M712
Luna Universal qPCR Master Mix	NEB	M3003L
Lipofectamine 3000	ThermoFisher Scientific	L3000008

**Experimental models: Cell lines**		

R01-09 ESC line	Yuri, S.et al.^25^	
rG104 rat ESC line	Isotani, A.et al.^46^	
Mesp1-KO ESCs	This study	
Mesp1/2-DKO ESCs	This study	

**Oligonucleotides**		

See Tables 1 and S2		

**Recombinant DNA**		

pSpCas9(BB)-2A-Puro (pX459) V2.0 plasmids	Addgene	#62988

**Software and algorithms**		

CrisperDirect	Naito, Y et al.^47^	https://crispr.dbcls.jp/
ImageJ-Fiji software	NIH images	https://imagej.nih.gov/ij/

### Experimental model and study participant details

#### Animals

All animal experiments were performed in accordance with the guidelines of “Regulations and By-Laws of Animal Experimentation at the Nara Institute for Science and Technology” and were approved by the Animal Experimental Committee at the Nara Institute of Science and Technology (approval nos. 1639 and 2109). A heterozygous Mesp1/2-KO (Mesp1/2-DHet) mouse line was established using the electroporation method as described previously with a small modification.^48^ Briefly, 8-week-old B6D2F1 female mice were treated with CARD HyperOva (Kyudo) and hCG (ASKA Animal Health) for superovulation and mated with C57BL/6N male mice. The zygotes were collected and placed in the electrode gap filled with 120 ng/μL Cas9 protein (Takara), 100 ng/μL crRNA1, 100 ng/μL crRNA2, and 300 ng/μL tracRNA (Fasmac) in Opti-MEM I solution (Gibco). Electroporation was performed at 30V (3 ms ON +/− 100 ms OFF) four times on CFB16-HB and LF501PT1-10 electrodes (BEX Co. Ltd.). After electroporation, the eggs were washed with M2 medium (SIGMA) and KSOM and incubated in KSOM until the eggs were transferred into pseudopregnant ICR females. The animal experiments in this study were performed in compliance with the ARRIVE guidelines.^49^ B6D2F1, ICR, and Wistar-Imamichi (WI) rats were purchased from Japan SLC, Inc. The crRNA1, crRNA2 and tracRNA sequence are shown in Table S1.

#### Cell culture

To establish Mesp1-KO and Mesp1/2-DKO ESCs, we used R01-09 ESC line, which was established from male blastocyst crossing mouse line of 129X1 and R01 in our previous study.^25^ The oligo DNAs for the target sequences of *Mesp1* and *Mesp2* (Table S1) were inserted into the pSpCas9(BB)-2A-Puro (pX459) V2.0 plasmids,^50^ which was given by Feng Zhang (Addgene, plasmid # 62988). All oligonucleotides were designed using the CrisperDirect website to identify specific target sites.^47^ The constructed plasmids were transfected into R01-09 ESCs using Lipofectamine 3000 (Thermo Fisher Scientific). The transfected cells were cultured for 2 days after transient treatment with 1 μg/mL puromycin (Sigma). Then, they were passaged for clonal culture. ESC colonies were subjected to PCR genotyping and sequencing. The ESCs were cultured on gelatin and MEF (mouse embryonic fibroblast) coated dish with N2 (Gibco) and B27 medium (Gibco) supplemented with 3 μM CHIR99021 (AXON), 1.5 μM CGP77675 (SIGMA), and mouse LIF (NPO in Osaka University) (N2B27-a2i/L medium).^51^^,^^52^ Rat ESC (rG104) were established from male blastocyst in previous study^46^ and were cultured on gelatin (Sigma) or Matrigel (Corning) and MEF coated dishes with N2B27 medium supplemented with 3 μM CHIR99021, 1.5 μM PD0325901 (Wako), mouse LIF, and human LIF (Sigma) (N2B27-2i/L medium).^53^ The quality of rat ESCs (rG104) was previously confirmed using teratoma formation assay or germline transmission.^46^^,^^54^ These cell lines were not contaminated with mycoplasma. Details of the cell sources are listed in key resources table.

### Method details

#### Genotyping

The primers for detecting the Mesp1-KO and Mesp1/2-DKO genotypes are shown in Table S1. DNA fragments were amplified using GoTaq (Promega) for 40 cycles to detect null or WT alleles under the following conditions: 94°C for 30 s, 60°C for 30 s, and 72°C for 60 s.

#### ESCs injection

ICR or Mesp1/2-DHet female mice (aged 8–10 weeks) were treated with CARD HyperOva and hCG for superovulation and mated with ICR or Mesp1/2-DHet male mice, respectively. The two-cell-stage embryos were collected from the female oviducts 42–46 h after hCG injection using the flush-out method. The collected two-cell stage embryos were incubated in KSOM medium at 37°C under 5% CO_2_ conditions until injection was performed. The rBC assay was performed as described previously.^25^ In total, 6–8 Mesp1-DKO or Mesp1/2-DKO ESCs were injected into ICR embryos at the 8-cell stage. The intraspecies BC method with mouse was performed by injecting 6–8 R01-09 ESCs into embryos obtained from Mesp1/2-DHet mice intercrossed at the E3.5 stage. For the interspecies BC method, four cells of the rG104 ESC line were injected into embryos obtained from the Mesp1/2-DHet mouse mating at E3.5 stage. The embryos were transferred into the uterus of E2.5- or E3.5-pseudopregnant ICR mice. For injecting rat ESCs into rat embryos, WI rats were superovulated with PMSG and hCG and the embryos were collected. Then, 4–6 rat ESCs were injected into the embryos, which were transferred into the uterus of E3.5 pregnant WI rats. The embryos or fetuses were dissected at E12.5, E14.5. The offspring were analyzed at 8 weeks of age for the mouse experiment and at E16.5 for the rat experiment. The chimeras were analyzed for RFP or GFP signals under a fluorescent stereomicroscope (Leica; MZFL III).

#### Flow cytometry analysis and fluorescence-assisted cell sorting

Chimeric embryos were recovered at the E12.5 or E14.5 stages. Tail, forelimb, heart, kidney, lung, stomach, and intestine samples were incubated with 0.25% trypsin/EDTA for 10 min at 37°C. After pipetting to dissociate the tissue, 10% FBS in phosphate buffered saline (PBS) was added, and samples were filtered through a 37-μm mesh. The FL1 detector on Accuri (BD Biosciences) or the FITC detector on CytoFLEX (Beckman Coulter) was used to detect the GFP+ population. The FL3 detector on Accuri or the ECD detector on CytoFLEX S were used to detect RFP+ population. MA900 (SONY) was used to sort the RFP subpopulations and remove the RFP+ population from the R01-09 ESC-derived cells for genotype analysis.

#### RNA expression analysis

Total RNA was purified using TRIzol reagent (Thermo Fisher Scientific). cDNA was prepared using the SuperScript IV VILO Master Mix (Thermo Fisher Scientific). Quantitative RT-PCR analysis was performed with the Luna Universal qPCR Master Mix (NEB) to amplify the DNA fragments. The amplified DNA was detected using a LightCycler 96 (Roche). The species specificity of all primer sets was assessed using amplification and melting curves based on the qPCR results. The primers used for RT-PCR are listed in Table S2.

#### Immunocytochemistry and HE staining

Heart, kidney or lung at E12.5, E14.5 or P56 were fixed overnight at 4°C with 4% paraformaldehyde (PFA) in PBS (−) (Nacalai). After washing with PBS (−), the tissues were immersed in 10, 15, and 20% sucrose in PBS (−) and then in Tissue-TeK O.C.T compound (Sakura Finetek). After sectioning into 10-μm thick sections with a cryostat (Leica; NX70 or CM3050S), the slides were dried at 25°C, followed by washing with PBS (−). For HE staining, the slides were immersed in H_2_O for 3 min, treated with Mayer’s hematoxylin solution (WAKO) for 3 min, washed with H_2_O, and immersed in 0.3% eosin solution (WAKO) for 3 min. The slides were first washed with H_2_O, second with 70%, 90%, and 100% EtOH, and finally twice with xylene. Entellan was added to the slides and the samples were sealed with coverslips. The sections were observed under a dissection microscope (M165FC; Leica). The immunostaining methods have been described previously.^25^ The antibodies used in this study are listed in key resources table. The immunostained slides were observed under a laser confocal microscope (LSM710 and LSM900; Zeiss, Mica; Leica).

#### Skeletal preparation in tail

The tails of 8-week-old mice were removed and fixed in 100% EtOH for 24 h, followed by treatment with 100% acetone for 2 days to remove the adipose tissue. The tails were incubated with an Alcian blue solution containing 0.03% Alcian blue (Nacalai), 7% acetic acid, and 56% EtOH. After rinsing twice with 70% EtOH and once with 100% EtOH, the tails were placed in 1% KOH at room temperature for 4 h at 25°C. The specimens were stained with Alizarin red solution containing 0.025% Alizarin red and 1% KOH for 24 h. The specimens were sequentially treated with 25%, 50%, 75%, and 100% glycerol and observed under a microscope (Leica; M165FC).^55^

#### Whole mount NBT/BCIP staining in lung

Lung samples were fixed in 4% PFA in PBS (Nacalai) for 1 h and washed once in PBS and twice with water. The lungs were incubated in NBT/BCIP Ready-to-Use (Nacalai) solution until a clear staining pattern appeared, followed by washing twice with water. The branching tips of the lungs were counted under a bright-field stereomicroscope (Leica; MZFL III).^56^

### Quantification and statistical analysis

All values pertaining to the relative fold changes of the flow cytometry and quantitative RT-PCR data are expressed as mean ± standard deviation from at least triplicate experiments. The Student’s t test was used for unpaired comparisons. The results at *p* < 0.01 were considered statistically significant.
