# Supplementary material for: Blastocyst complementation-based rat-derived heart generation reveals cardiac anomaly barriers to interspecies chimera development
Source: iScience. 2024 Nov 18;27(12):111414. doi: 10.1016/j.isci.2024.111414 (PMC11647242; doi:10.1016/j.isci.2024.111414)
Supplement: Document S1. Figures S1–S5 and Tables S1 and S2 [file mmc1.pdf]

iScience, Volume *27*

Supplemental information

Blastocyst complementation-based rat-derived heart generation reveals cardiac anomaly barriers to interspecies chimera development

Shunsuke Yuri, Norie Arisawa, Kohei Kitamuro, and Ayako Isotani

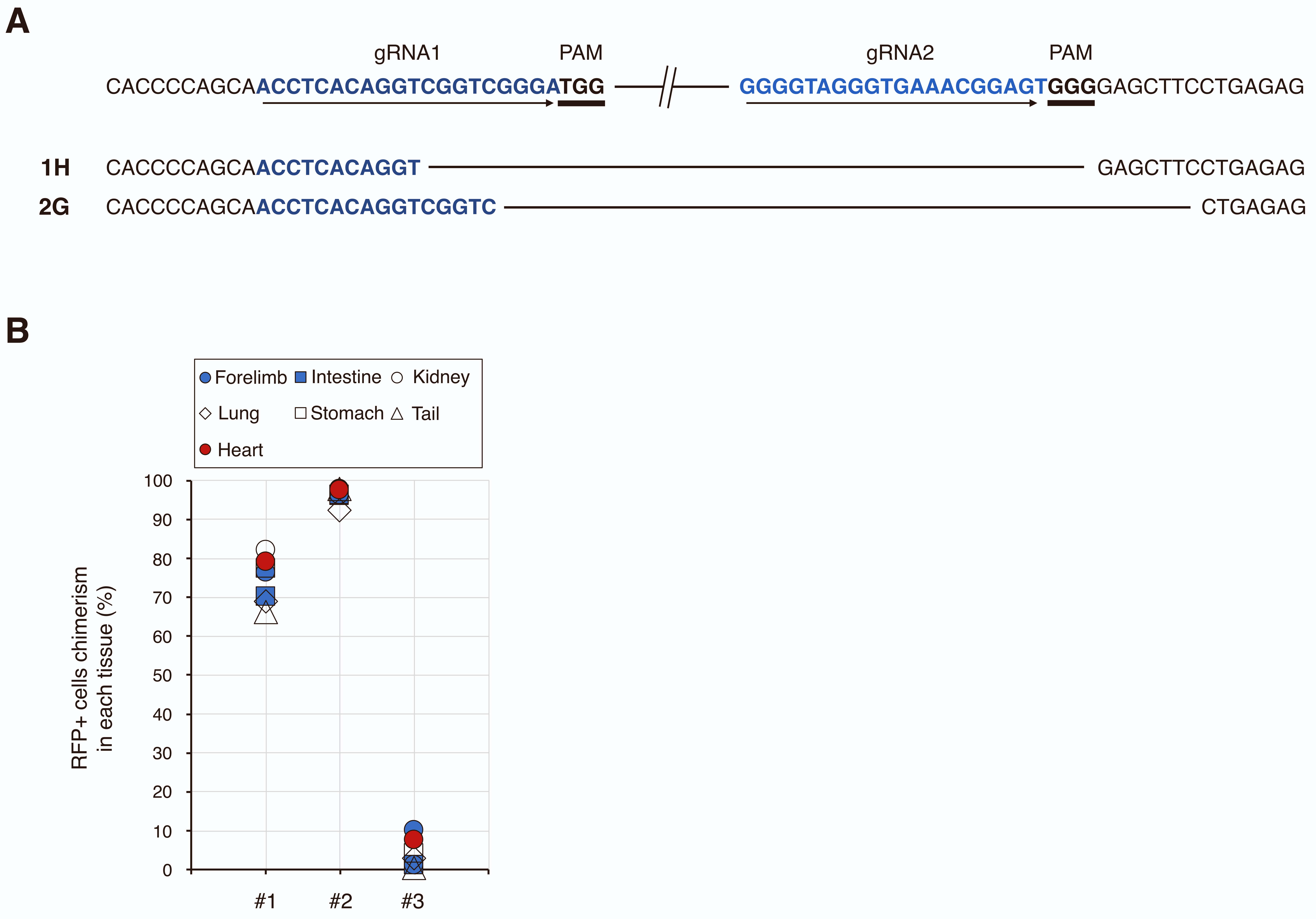

Figure S1. Analysis of Mesp1-KO model, related to Figure 1.

(A) Mutation patterns of the obtained Mesp1-KO ESC lines

(B) Flow cytometry results for RFP-expressing Mesp1-KO cells in forelimb tail, kidney, lung, stomach, and intestine

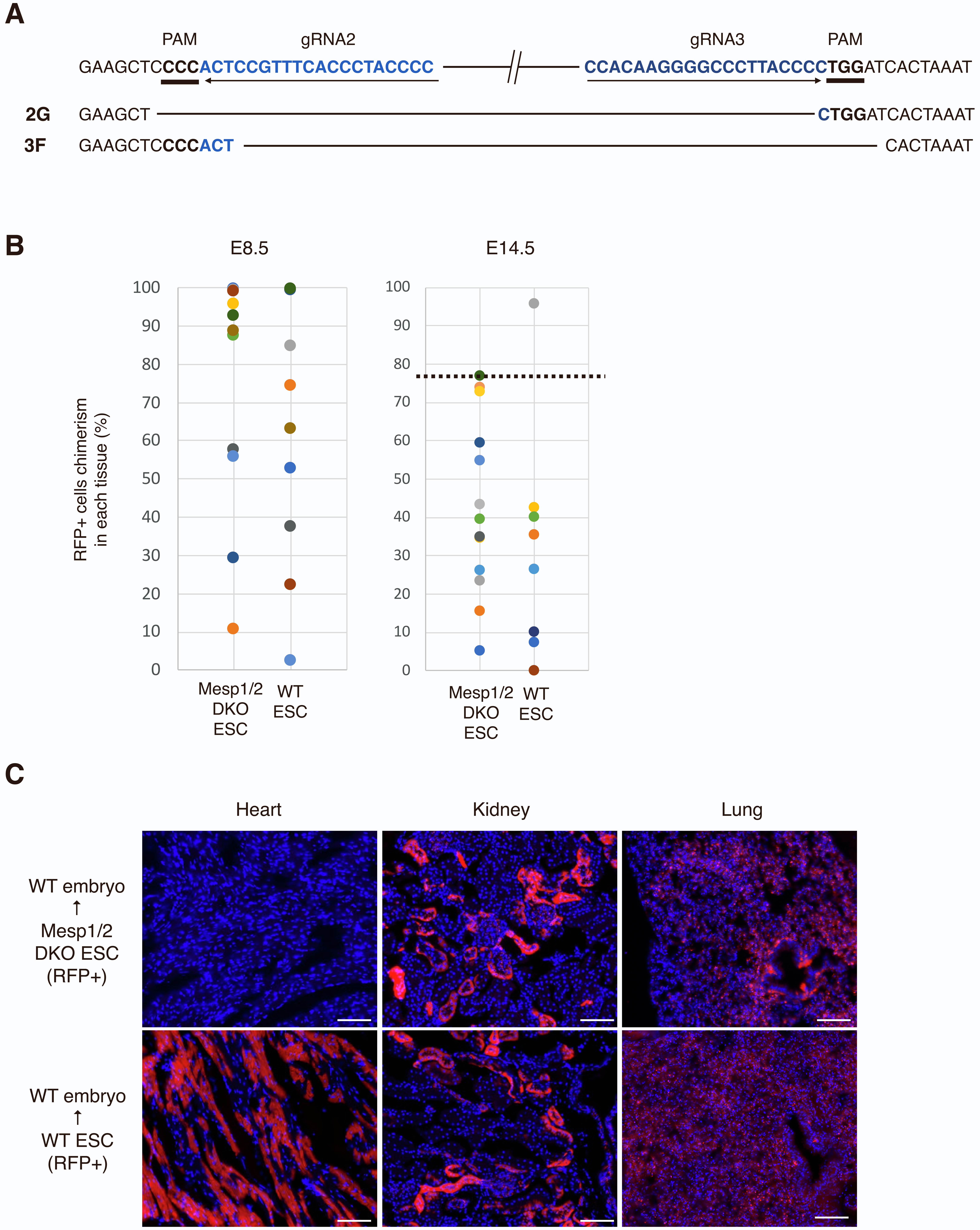

Figure S2. Analysis of Mesp1/2-DKO model using rBC method, related to Figure 2, 3 and 4.
(A) Mutation patterns of the obtained Mesp1/2-DKO ESC lines
(B) Flow cytometry results for RFP-expressing Mesp1/2 DKO cells at E8.5 and E14.5
(C) Representative sectioning images of heart, kidney and lung in Mesp1/2-DKO ESCs+WT and WT ESCs+WT chimeras at P56. Four non-overlapping random fields were analyzed (Scale: 100μm).

A

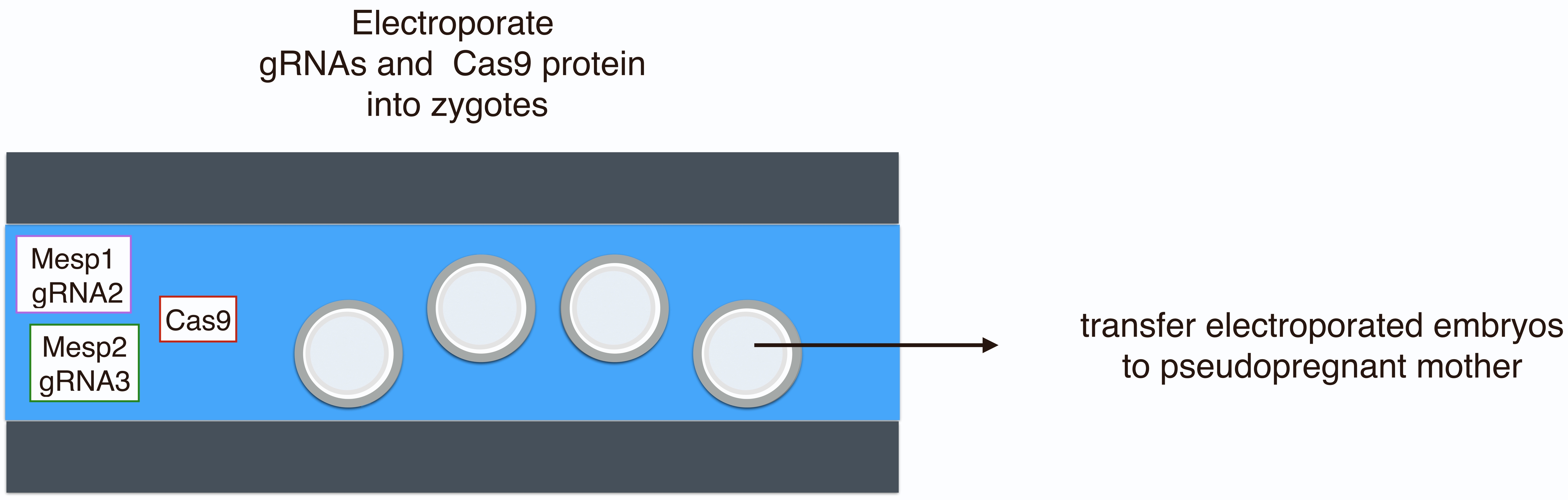

B

Electroporated embryo	Pups	Mutation on gRNA2	Mutation on gRNA3	Delete 21kb region
88	8	7	3	1

C

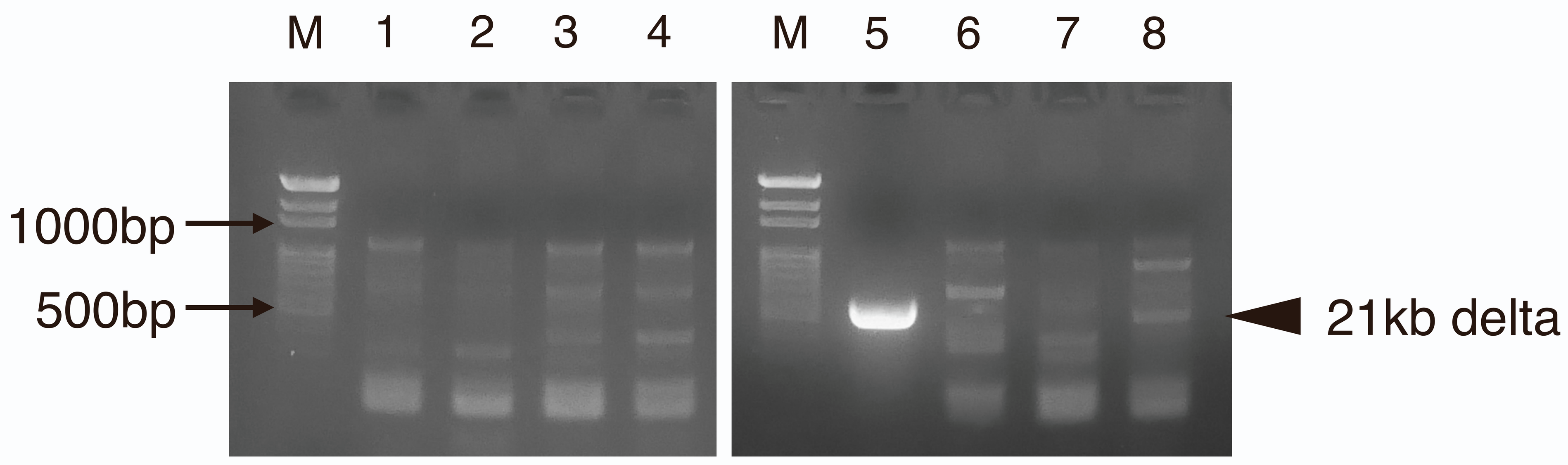

D

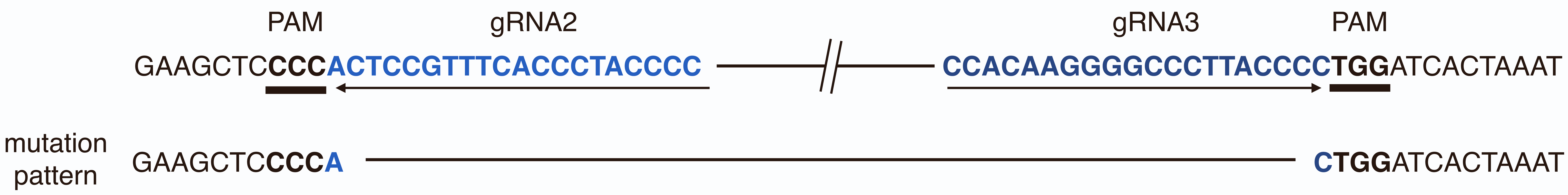

E

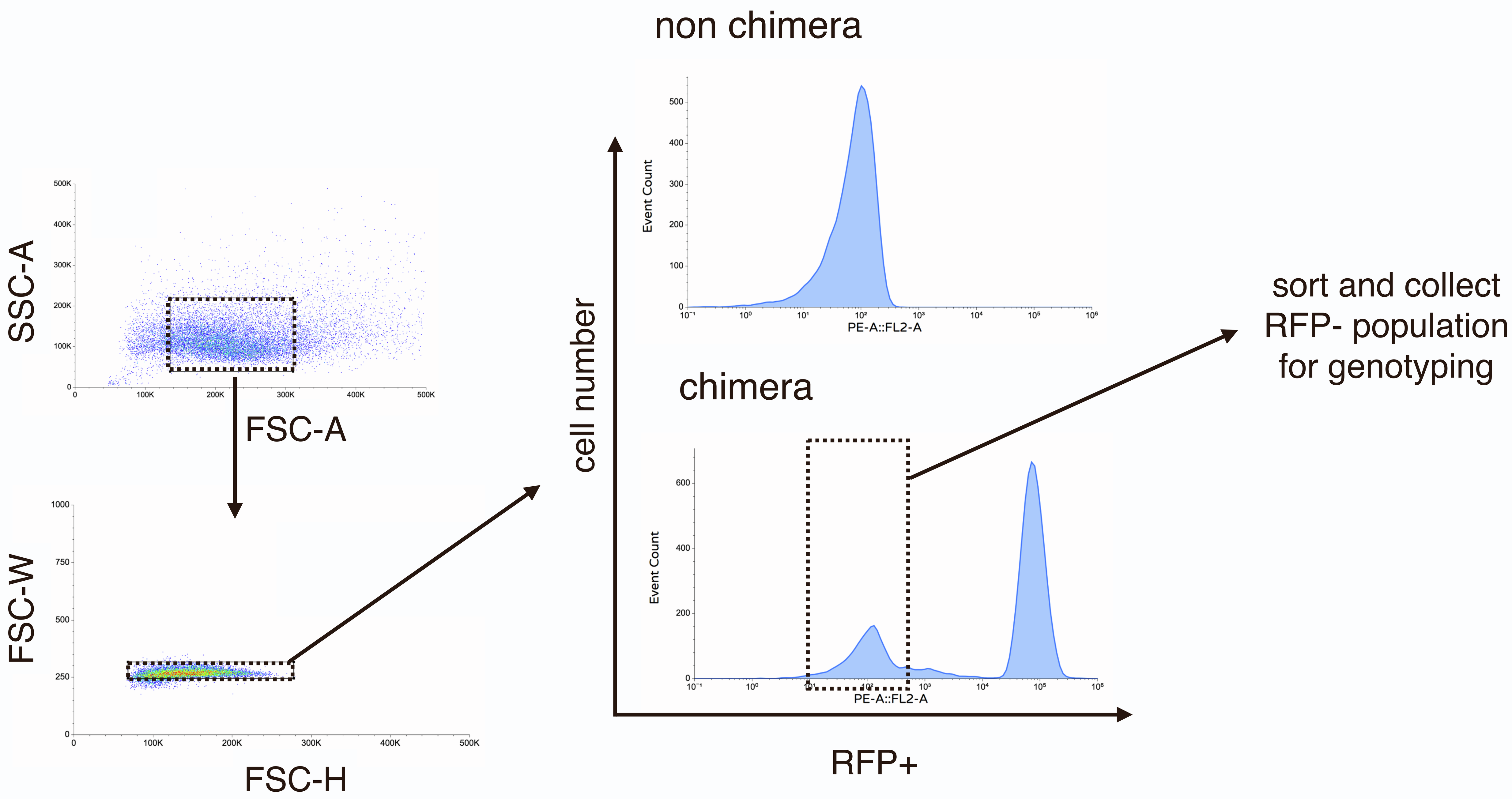

Figure S3. Analysis of Mesp1/2-DKO model using BC method, related to Figure 5

(A) Schematic of the electroporation method

(B) Results of the electroporation method

(C) Genotype result of mice obtained using electroporation method

(D) Mutation patterns of the obtained Mesp1/2-DHet mouse line

(E) Genotype strategy of obtaining chimeras by crossing Mesp1/2-DHet with Mesp1/2-DHet using the intraspecies blastocyst complementation method

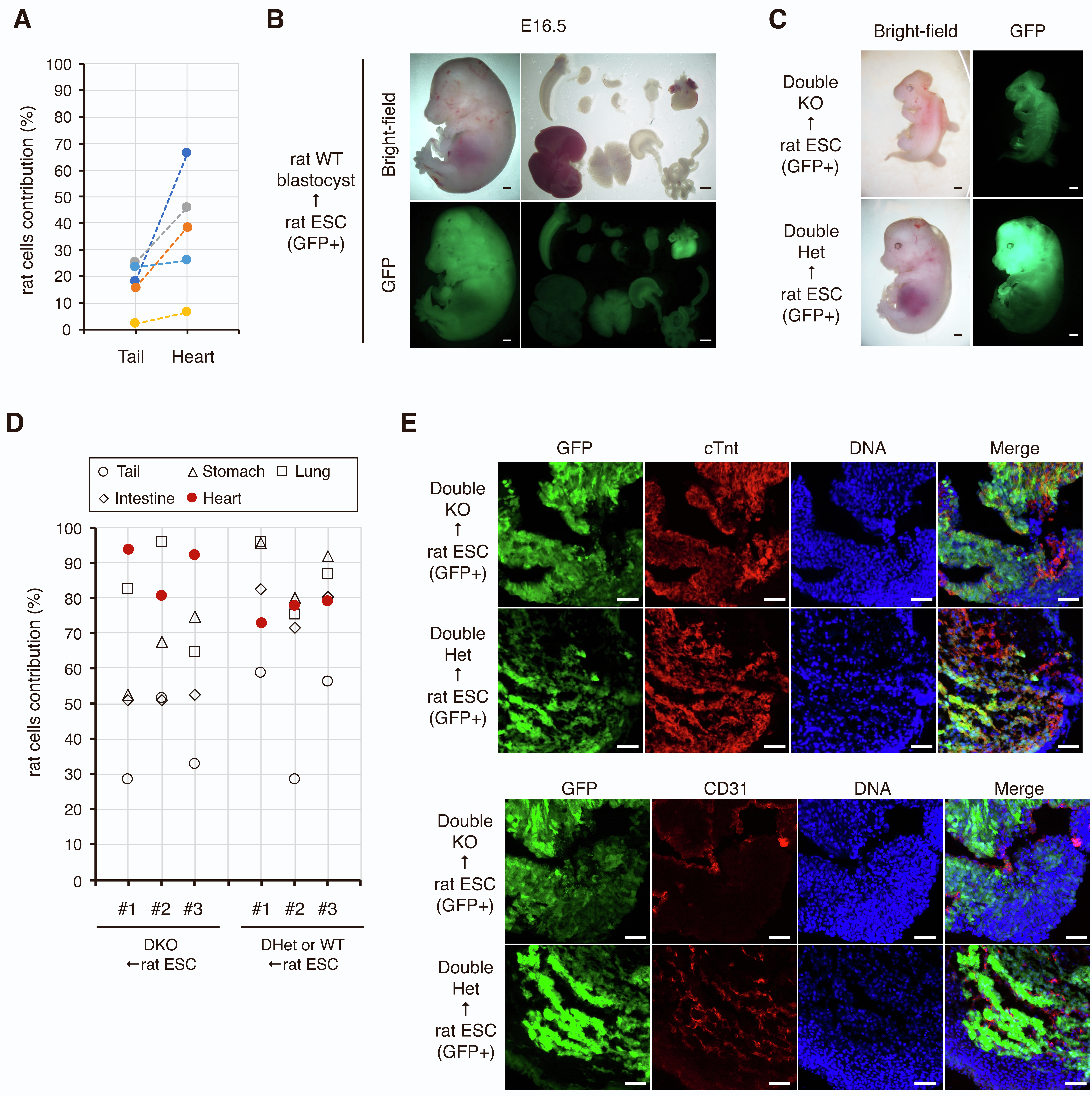

Figure S4. Analysis of mouse–rat chimera in Mesp1/2-DKO model, related to Figure 6.

(A) Flow cytometry analysis of tail and heart of rat ESCs+WT chimeras at E14.5. (n=5)

(B) Representative images of rat ESCs+WT rat chimera embryos at E16.5. The chimera showed 78.4% rat ESCs derived cells in the tail and 79.3% in the heart. (Scale bar: 1 mm)

(C) Representative images of rat chimera embryo from the Mesp1/2-DKO model and non-Mesp1/2-DKO model at E14.5. (Scale bar: 1 mm)

(D) Flow cytometry analysis of organs (tail, stomach, lung, intestine, and heart) in chimeras from rat ESCs+Mesp1/2-DKO or rat ESCs+non-Mesp1/2-DKO.

(E) Representative immunostaining image of cTnt and CD31 in the heart of rat ESCs+Mesp1/2-DKO or rat ESCs+non-Mesp1/2-DKO chimera. Three non-overlapping random fields were analyzed (Scale bars: 100 µm).

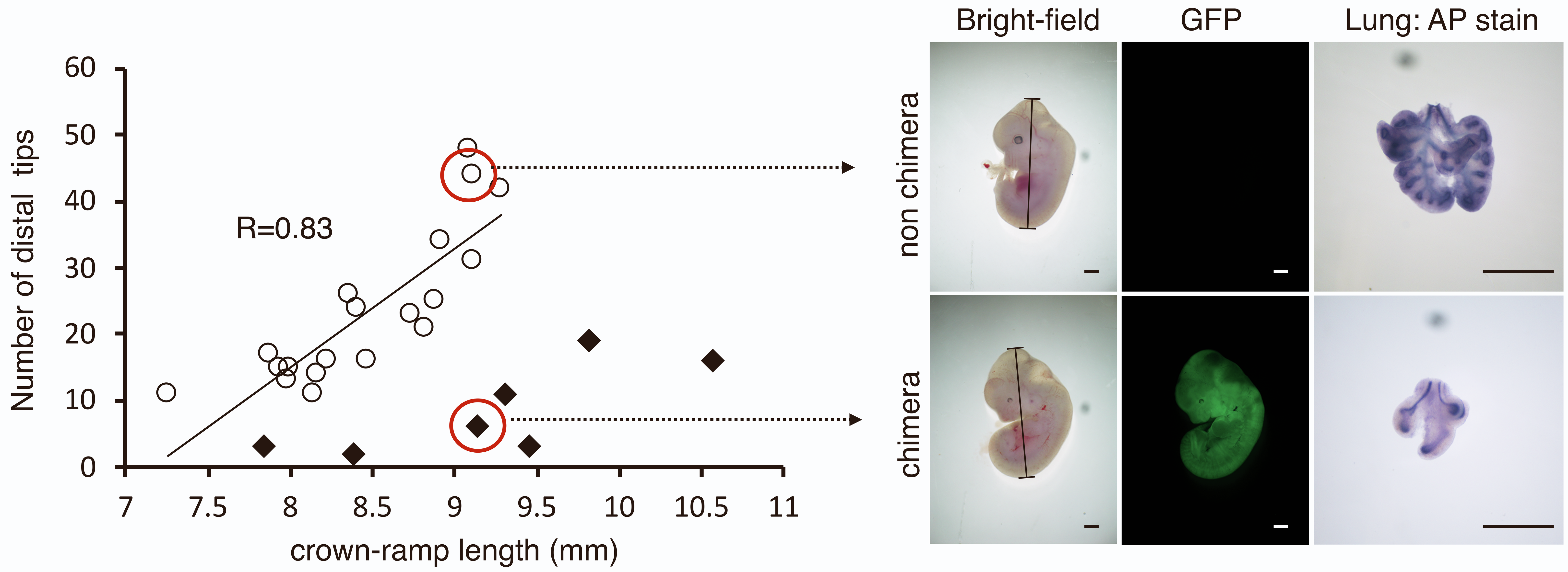

Figure S5. Analysis of mouse–rat chimera in lungs of non-Mesp1/2-DKO model, related to Figure 7.
Correlation between crown ramp length and number of distal tips in the lungs. Lung epithelial cells were stained with AP (alkaline phosphatase) and counted. Each point represents individual measurements in the scatter plot. Open circles represent data for mice, with a correlation coefficient (R) of 0.83 (n=18). Black squares show the result for mouse–rat chimeras with higher rat contribution (33.2–68.9% in tail) (n=7). Images showing embryos and lungs with AP staining in non-chimeras and chimeras with similar crown ramp lengths. (Scale bar: 1 mm)

Table S1. Oligonucleotide information, related to Method.

ID	oligo name	sequence
#1	crRNA1 for Mesp1	GGGGUAGGGUGAAACGGAGUguuuuagagcuaugcuguuuug
#2	crRNA2 for Mesp2	CCACAAGGGGCCCUUACCCCguuuuagagcuaugcuguuuug
#3	TracRNA	aaacagcauagcaaguuaaaauaaggcuaguccguuaucaacuugaaaaaguggcaccgagucggugcu
#4	Mesp1-sgRNA1-F	caccACCTCACAGGTCGGTCGGGA
#5	Mesp1-sgRNA1-R	aaacTCCCGACCGACCTGTGAGGT
#6	Mesp1-sgRNA2-F	caccGGGGTAGGGTGAAACGGAGT
#7	Mesp1-sgRNA2-R	aaacACTCCGTTTCACCCTACCCC
#8	Mesp2-sgRNA2-F	caccCCACAAGGGGCCCTTACCCC
#9	Mesp2-sgRNA2-R	aaacGGGGTAAGGGCCCCTTGTGG
#10	Mesp1-KO check F	ACTGCCATTGGGACAGATGA
#11	Mesp1-KO check R	CCCAAGCCTTACATGGCTCA
#12	Mesp1-WT check F	AGTCCTGGATCCTGAGTCCC
#13	Mesp1-WT check R	AACAGCGCCACAATCAACAC
#14	Mesp1/2-DKO check F	TGTGAAAGAGCCCTACCGC
#15	Mesp1/2-DKO check R	AGCGATCAACAGGTCCCTCA
#16	Mesp1/2-WT check F	CAAGCTGTGGTGACTGTTGC
#17	Mesp1/2-WT check R	CTCGTCCTCAGATTCGTCCG

Table S2. Primer information for RT-PCR analysis, related to Method and Figure 6.

ID	oligo name	sequence
#1	RT-mGapdh F	CATTTGCAGTGGCAAAGTGGAG
#2	RT-mGapdhR	CGTCAGATCCACGACGGAC
#3	RT-mouse Myl2 F	GAAAGCCAAGAAGCGGATAGAA
#4	RT-mouse Myl2 R	GTTGAGAATGGTCTCTTCAGGA
#5	RT-mouse Myl7 F	TTCCGGAGGAAGAGCTGGAC
#6	RT-mouse Myl7 R	GTCAGCGCAAACAGTTGCTCT
#7	RT-mouse Aldh1a2 F	CCACCCGGAGTCGTCAATATC
#8	RT-mouse Aldh1a2 R	TCTTCCCTCCGAGTTCCAGG
#9	RT-mouse Pecam F	TGACTTCCAGACTCTCGAGG
#10	RT-mouse Pecam R	TACTCGACAGGATGGAAATCAC
#11	RT-mouse Postn F	TTGAGATAGGGTGCGAAGGG
#12	RT-mouse Postn R	GCTTCAGAGAGGATGCCAAG
#13	RT-rGapdh F	CTTCTCTTGTGACAAAGTGGAC
#14	RT-rGapdh R	ATGTCAGATCCACAACGGAT
#15	RT-rat Myl2 F	GAAAGCCAAGAAGAGGTTAGAG
#16	RT-rat Myl2 R	GTTGAGAATGGTCTCCTCC
#17	RT-rat Myl7 F	GAGACGGAATCATCTGCAAG
#18	RT-rat Myl7 R	TCGGGTCAAACATTCGGAAA
#19	RT-rat Aldh1a2 F	TCACGAGACATGAGCCCATC
#20	RT-rat Aldh1a2 R	CTGGCAGAATGTTGACGACC
#21	RT-rat Pecam F	AACTTCCAGACCGTCCAGAA
#22	RT-rat Pecam R	CACCGAAGCACCATTTCATC
#23	RT-rat Postn F	TTGAAATAGGGTGTGAGGGA
#24	RT-rat Postn R	CTTCAGAGAAGACGCCAAC
